# The effect of prebiotic fortified infant formulas on microbiota composition and dynamics in early life

**DOI:** 10.1038/s41598-018-38268-x

**Published:** 2019-02-21

**Authors:** Klaudyna Borewicz, Maria Suarez-Diez, Christine Hechler, Roseriet Beijers, Carolina de Weerth, Ilja Arts, John Penders, Carel Thijs, Arjen Nauta, Cordula Lindner, Ellen Van Leusen, Elaine E. Vaughan, Hauke Smidt

**Affiliations:** 1 Laboratory of Microbiology, Wageningen University & Research, Stippeneng 4, 6708 WE Wageningen, The Netherlands; 2 Laboratory of Systems and Synthetic Biology, Wageningen & Research University, Stippeneng 4, 6708 WE Wageningen, The Netherlands; 3 Behavioral Science Institute, Radboud University, Montessorilaan 3, 6525 HR Nijmegen, The Netherlands; 4 Department of Cognitive Neuroscience, Donders Institute for Brain, Cognition and Behaviour, Radboud University Medical Center, Kapittelweg 29, 6525 EN Nijmegen, The Netherlands; 5 Department of Epidemiology, Maastricht University, Care and Public Health Research Institute, Minderbroedersberg 4-6, 6211 LK Maastricht, The Netherlands; 6 CARIM School for Cardiovascular Diseases, Universiteitssingel 50 6229ER Maastricht; and Maastricht Center for Systems Biology (MaCSBio), Universiteitssingel 60, 6229 ER Maastricht, The Netherlands; 7 Department of Medical Microbiology, Maastricht University Medical Center, P. Debyelaan 25, 6229HX Maastricht, The Netherlands; 8 NUTRIM School for Nutrition, Toxicology and Metabolism, Universiteitssingel 40, 6229 ER Maastricht, The Netherlands; 9 FrieslandCampina, Stationsplein 4, 3818 LE Amersfoort, The Netherlands; 10 Sensus B.V. (Royal Cosun), Borchwerf 3, 4704 RG Roosendaal, The Netherlands

## Abstract

Gastrointestinal (GI) microbiota composition differs between breastfed and formula-fed infants. Today’s infant formulas are often fortified with prebiotics to better mimic properties of human milk with respect to its effect on GI microbiota composition and function. We used Illumina HiSeq sequencing of PCR-amplified 16S rRNA gene fragments to investigate the composition of faecal microbiota in 2–12 week old infants receiving either breastmilk, infant formulas fortified with prebiotics, or mixed feeding. We compared these results with results from infants fed traditional formulas used in the Netherlands in 2002–2003, which contained no added prebiotics. We showed that today’s formulas supplemented with either scGOS (0.24–0.50 g/100 ml) or scGOS and lcFOS (at a 9:1 ratio; total 0.6 g/100 ml) had a strong bifidogenic effect as compared to traditional formulas, and they also resulted in altered patterns of microbial colonisation within the developing infant gastrointestinal tract. We identified three microbial states (or developmental stages) in the first 12 weeks of life, with a gradual transition pattern towards a bifidobacteria dominated state. In infants receiving only fortified formulas, this transition towards the bifidobacteria dominated state was accelerated, whereas in infants receiving mixed feeding the transition was delayed, as compared to exclusively breastfed infants.

## Introduction

Microbial colonisation of an infant’s gastrointestinal (GI) tract starts before or at birth and progresses in a step-wise fashion during the postnatal period^1–3^. Many environmental factors may affect GI microbiota composition and its development during early life^4–8^. These early life exposures and associated GI microbiota perturbations have been linked with changes in immune development leading to potentially serious and lifelong health effects. For example, earlier studies suggested that infants fed formula were at higher risk of nutritional deficiencies, asthma, atopy, obesity, developing metabolic syndrome, coeliac disease, diabetes and other diseases, as compared to breastfed infants^9^. Some of the early life exposures cannot be avoided, however, the use of formula feeding is, at least in some cases, a choice made by the parents^9^. Over the last century, when formula feeding became more popular worldwide^10^, intensive research led to developing infant formulas that are increasingly similar to human milk with regard to nutrient composition and function. However, they are certainly not identical, and breastmilk with its complex composition still remains the golden standard for infant nutrition^10,11^. Human milk contains a wide range of compounds that modulate the infant intestinal microbiota^9,12^. One of the prevalent and important groups of components are the human milk oligosaccharides (HMOs) that have unique nutritional and functional properties^13^. The prebiotic function of breastmilk as source of growth factors for the “bifidus flora” was identified more than a hundred years ago^14^. Various studies since then confirmed that the GI microbiota of breastfed infants is dominated by bifidobacteria, as compared to formula fed infants, a fact which has been most often attributed to the presence of HMOs in the human milk, and their lack in infant formulas^15,16^. Taking into account both the wide use of infant formulas and the growing evidence for the importance of GI microbiota for health throughout life, it became clear that the functional prebiotic properties of infant formulas needed to be addressed. Thus, formulas nowadays are often fortified with prebiotics. In European countries these prebiotics include mostly short chain galacto-oligosaccharides (scGOS) alone, or in a mixture with a chicory root derived inulin containing long chain fructo-oligosaccharides (lcFOS)^17,18^. Prebiotics mimic the bifidogenic effect of HMOs in human milk and have been associated with improved immunity, bowel function and other health benefiting effects in infants^13,19,20^. However, the exact effect of these functional alternatives on the GI tract microbial ecosystem, most importantly with respect to the dynamics of bacterial colonisation in early life, are not yet well understood and should be investigated.

Here we present the results of a longitudinal study in which we compared the colonisation patterns of breastfed and formula fed infants, including a group of mixed fed infants. We assessed the microbiota composition in faecal samples from two, six and 12 weeks old infants born between years 2015–2016 and receiving commercial formulas fortified with GOS and/or FOS. We compared those results with the faecal microbiota composition of one month old infants born in 2002–2003 and fed commercial infant formulas purchased during those years. In both studies, infants received formulas that were available on the Dutch market at the time the samples were collected. We show that the new prebiotic supplemented formulas have a bifidogenic effect on infant GI microbiota, however, they also result in altered dynamics of bacterial colonisation during the first 12 weeks of life as compared to breastfed infants.

## Results

A total of 443 samples were included in the analyses: 204 samples (from 74 infants) and 239 samples (from 239 infants) from the BINGO and KOALA cohort, respectively. A total of 28,955,759 sequencing reads were obtained from the BINGO cohort samples, with per sample counts ranging from 5,215 to 721,990 (Mean = 141,940; *SD* = 126,570), with 95% of samples having at least 20,000 reads. Sequencing of the KOALA cohort samples resulted in a total of 30,132,625 sequencing reads ranging from 1,380 to 448,285 per sample (Mean = 126,078, *SD* = 84,356), with 95% of samples having at least 25,000 reads. Taxonomic classification of OTUs was done for the complete sample set using the NG-Tax pipeline against a customised SILVA database^21^, and it resulted in detection of five different phyla, namely Actinobacteria, Bacteroidetes, Firmicutes, Proteobacteria, and Verrucomicrobia. At the genus level, the most abundant taxa were *Bifidobacterium, Bacteroides, Streptococcus, Escherichia-Shigella*, and an unassigned genus within the family Enterobacteriaceae (Table S1).

Being overall the most abundant genus-level taxonomic group in infant faecal microbiota, we first assessed potential differences in relative abundance of bifidobacteria with age and different feeding modes. In the BINGO cohort faecal samples were collected between years 2015–2016 from infants at two, six and 12 weeks of age, and most commercial infant formulas used in the BINGO cohort contained prebiotics. In the BINGO cohort we observed an age related increase in the average relative abundance of bifidobacteria in the BF infants (Kruskal-Wallis; p = 0.01). In contrast, in FF and MF infants from the BINGO cohort the relative abundance of bifidobacteria fluctuated, and the differences were not significant between different age groups (Kruskal-Wallis: p > 0.05). In the BINGO cohort, MF was associated with lower relative abundance of bifidobacteria, as compared to FF infants (Kruskal-Wallis: p = 0.0078). In the KOALA cohort, in which infants were born between years 2002–2003, most commercial formulas did not contain added prebiotics. In this cohort, MF resulted in significant reduction in the relative abundance of bifidobacteria as compared to BF (Kruskal-Wallis: FDR = 0.05, p = 0.00078), but not in comparison to the FF group (Kruskal-Wallis: p > 0.05).

**Figure 1:**
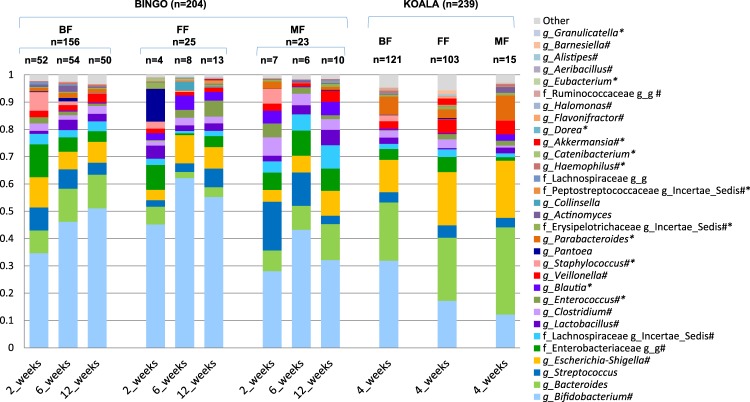
Average relative abundance of genus level taxa in faeces of infants included in the BINGO and KOALA cohorts that were either breastfed (BF), formula fed (FF) or fed both breastmilk and formula (mixed fed, MF). When the taxonomic assignment could not be made at genus level, the lowest classifiable taxonomy assignment is used instead and unidentified genus is indicated with “g_g”. Taxa that significantly differ (Wilcoxon test, FDR < 0.05) in their relative abundance between BF and FF infants are indicated with *(BINGO) and ^#^(KOALA).

To better relate the results from the two study cohorts we focused our comparisons on the BINGO study infants at six weeks of age. FF infants in the BINGO cohort received prebiotic fortified formulas and showed higher average relative abundance of bifidobacteria as compared to BF infants (62% in FF vs. 46% in BF.; difference not statistically significant), whereas in the KOALA cohort the abundance of bifidobacteria in the FF infants was significantly lower (Wilcoxon test, FDR < 0.05) than that in the corresponding BF infants from the KOALA study (17% in FF vs. 32% in BF) (Fig. 1). In addition, OTU level analyses revealed that in both study cohorts the same three *Bifidobacterium* OTUs (denoted as L1, L2 and B1) were most predominant in the faeces of the one month old infants (Fig. S1, Table S2). Remarkably, while in the KOALA cohort formula feeding and breastfeeding resulted in significantly different distributions in all three major bifidobacterial OTUs, in the BINGO cohort the relative abundance of the most abundant *Bifidobacterium* OTU L2 was not significantly different between infants fed breastmilk, formula or both. In contrast, *Bifidobacterium* OTU L1 increased significantly in formula fed infants in both cohorts. When infants received mixed feeding (breastmilk and formula), there were no significant differences in the main bifidobacterial OTUs as compared to breastfed infants in the BINGO cohort, whereas in the KOALA cohort there was a significant decrease in the relative abundance of *Bifidobacterium* OTU L2 (Fig. S1).

To identify genus level taxa that were significantly different between BF and FF infants in each study cohort (Fig. 1) we used the Wilcoxon test. In the BINGO cohort 12 genus level groups differed significantly between BF and FF infants when all age groups were analysed together (FDR < 0.05, p < 0.0057; Fig. 1), but at six weeks of age only *Blautia* (an adult-like taxon) was identified as significantly enriched in the FF group (FDR = 0.0001, p < 0.001). In infants included in the KOALA cohort, the relative abundances of 19 genus level taxa were statistically different between both feeding types (FDR < 0.05, p < 0.0058; Fig. 1).

Formula feeding significantly (FDR < 0.05) increased relative abundance of *Akkermansia, Enterococcus*, Peptostreptococcaceae *Incertae Sedis*, and Erysipelotrichaceae *Incertae Sedis*, and significantly decreased relative abundance of *Staphylococcus* and *Haemophilus*, as compared to the corresponding BF groups in both study cohorts. In addition, in the BINGO cohort only, formula feeding significantly increased relative abundance of *Blautia, Dorea, Granulicatella, Eubacterium, Catenibacterium*, and decreased relative abundance of *Parabacteroides* as compared to the BF group. In the KOALA cohort, formula feeding significantly increased relative abundance of *Barnesiella, Alistipes, Escherichia-Shigella, Veillonella, Flavonifractor, Clostridium*, Lachnospiraceae *Incertae Sedis*, and unidentified genera within the families Ruminococcaceae and Enterobacteriaceae, whereas it decreased *Bifidobacterium, Lactobacillus, Halomonas*, and *Aeribacillus* as compared to the BF group.

**Figure 2:**
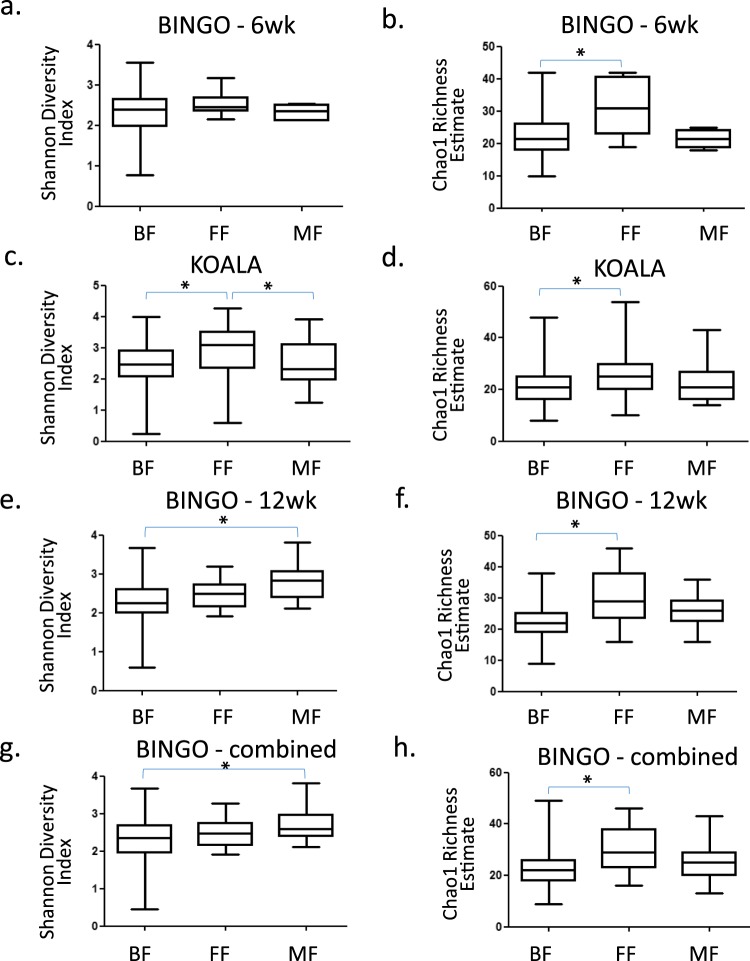
Alpha diversity of faecal microbiota in breastfed (BF), formula fed (FF) or mixed fed (MF) infant groups; (**a**) Bacterial diversity in the BINGO study infants at six weeks of age; (**b**) Bacterial richness in the BINGO study infants at six weeks of age; (**c**) bacterial diversity in the KOALA study infants at one month of age; (**d**) Bacterial richness in the KOALA study infants at one month of age; (**e**) Bacterial diversity in the BINGO study infants at 12 weeks of age; (**f**) Bacterial richness in the BINGO study infants at 12 weeks of age; (**g**) Bacterial diversity in the BINGO study infants at two, six, and 12 weeks of age combined; (**h**) Bacterial richness in the BINGO study infants at two, six, and 12 weeks of age combined. *Indicates statistically significant difference between groups (p < 0.05).

Alpha diversity estimates were compared between feeding modes and between different age groups of infants from the BINGO cohort (Fig. 2). There were no statistically significant differences in PD Whole Tree, Shannon Diversity Index or Chao1 Index between age groups. No differences between BINGO BF, FF, MF at individual time points or when three time points were combined were detected in PD Whole Tree estimates (p = 0.227) (data not shown). Shannon Diversity Index values in the BINGO cohort were significantly higher in MF infants as compared to BF infants (Shannon Diversity: p = 0.016; Fig. 2e) at week 12 only, or when all age groups were combined (Shannon Diversity; p = 0.0035; Fig. 2g). When Chao1 species richness index was used, FF infants had significantly higher richness than BF infants at six weeks (Chao1: p = 0.0085; Fig. 2b) and 12 weeks (Chao1: p = 0.0005; Fig. 2f) of age, and when age groups were combined (Chao1: p = 0.0002; Fig. 2h). The fact that differences in bacterial richness and diversity were only observed with Chao1 and Shannon indices but not with PD Whole Tree index suggests that those differences mostly concerned closely related taxa. The Shannon’s diversity index accounts for both abundance and evenness of the species present, whereas the PD Whole Tree index measures phylogenetically weighted species richness.

In the KOALA study cohort there was a statistically significant difference in diversity estimated with the Shannon Diversity Index between BF and FF infants (Shannon Diversity: p < 0.0001) and between FF and MF infants (Shannon Diversity: p = 0.035), but not between BF and MF infants (Fig. 2c), with the highest diversity detected in the FF infants. Comparison of alpha diversity based on the PD Whole Tree estimates showed no significant differences between infants receiving different feeding types (data not shown). Bacterial richness was significantly higher in the FF infants as compared to BF infants (Chao1: p < 0.0001; Fig. 2d). In general, both the faecal bacterial diversity and richness were higher in FF infants as compared to BF infants, whereas MF infants showed an intermediate phenotype.

**Figure 3:**
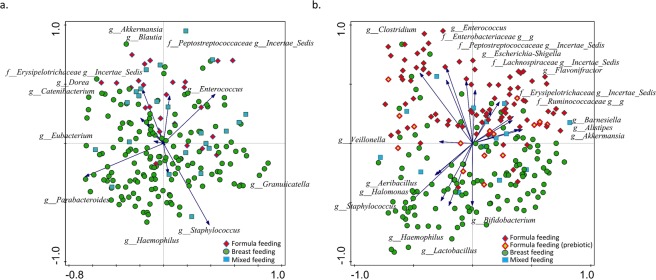
PCA analysis of log transformed genus level relative abundance data. Samples coloured by infant feeding type showing separation between different feeding modes. Microbial groups that significantly differ in relative abundance (Wilcoxon test, FDR < 0.05) between BF and FF groups are displayed; (**a**) BINGO study, (**b**) KOALA study.

In both BINGO and KOALA sample sets, Principle Component Analysis (PCA) revealed grouping of samples into FF and BF groups with the MF samples scattered in between (Fig. 3a,b). There was no separation of samples from the 17 FF infants in the KOALA cohort who received prebiotic fortified formula (p = 0.076). The separation of samples according to the feeding mode was more pronounced among the KOALA cohort infants, and the bacterial taxa driving the separation differed between the studies. Nevertheless, despite a much smaller FF group size in the BINGO cohort, the overall sample distribution pattern on the PCA plots was conserved between both studies.

**Figure 4:**
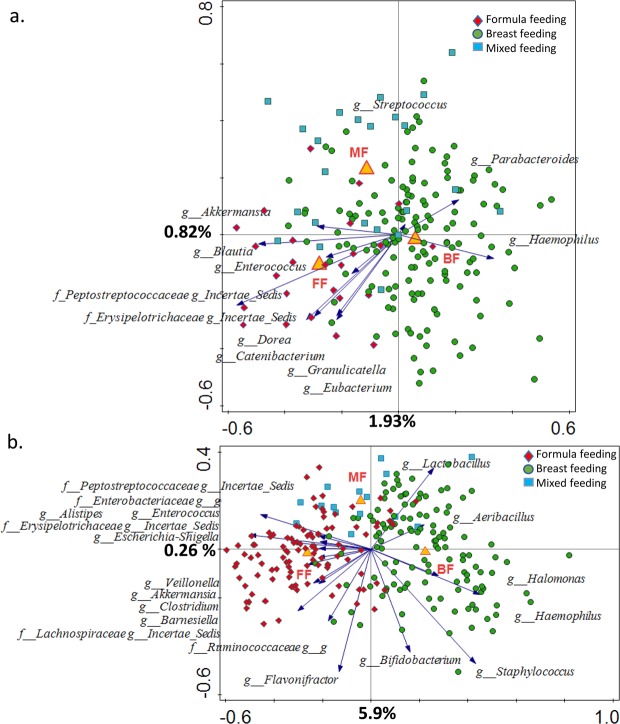
Partial RDA analysis with covariates, using the log transformed genus level relative abundances. Samples coloured by infant feeding type (BF, breastfeeding; FF, formula feeding; MF, mixed feeding) showing separation between different feeding modes. Microbial groups that significantly differ in relative abundance (Wilcoxon test, FDR < 0.05) between BF and FF groups are displayed; (**a**) BINGO study, (**b**) KOALA study.

Redundancy analysis (RDA) was used to assess the amount of variation in the microbial composition data, which could be explained by feeding mode and additional demographic factors recorded for the two different cohorts. These factors included place and mode of delivery, gender, and medication use (Fig. S2a,b). The interactive forward selection method identifies a subset of variables that best explain the variation in the data. In the BINGO cohort, when all samples were analysed together, age, feeding (BF, FF, MF), mode of delivery (C-Section, vaginal), place of delivery (home, hospital, clinic), and medication had a significant effect on microbiota composition (FDR < 0.05) and together explained 13.7% of variation. In the KOALA cohort, feeding and delivery mode were selected (FDR < 0.05) during interactive forward selection, and they together explained 9.5% variation. To investigate the residual effect of feeding mode separately, we repeated the RDA analysis with feeding mode as the main factor and all other factors selected during the interactive selection process as covariates. In both the BINGO cohort and in the KOALA cohort, the effect of feeding was significant (FDR < 0.05), and feeding explained 2.9%, and 6.2% of the residual variation, respectively (Fig. 4a,b).

**Figure 5:**
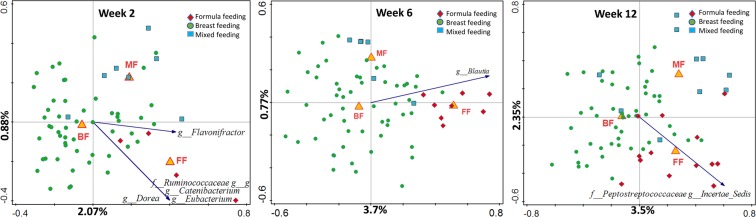
RDA analysis with covariates, using the log transformed genus level relative abundance data at each time point in the BINGO cohort. Samples coloured by infant feeding type (BF, breastfeeding; FF, formula feeding; MF, mixed feeding) showing separation between different feeding modes. Microbial groups that significantly differ in relative abundance (Wilcoxon test, FDR p < 0.05) between BF and FF groups at each time point are displayed.

We then applied the same approach at each time point separately to investigate in more detail the effect of different factors (feeding, place and mode of delivery, gender, and medication use) in the BINGO cohort data (Fig. 5). The interactive selection showed that both the place and the mode of delivery had a significant effect on microbiota at two weeks of age (FDR < 0.05). At six weeks, the effects of mode of delivery and feeding were significant, and at 12 weeks only the effect of feeding was significant. When delivery mode was set as covariate, feeding could explain 4.5% of the residual variation in the microbiota composition at week six and increased to 5.9% at week 12.

In both studies, RDA analyses showed that breastfeeding and formula feeding resulted in differences in microbiota composition (FDR < 0.05). In contrast, the effect of mixed feeding was not significant in the KOALA cohort and in the six week old infants in the BINGO cohort. However, when samples from all time points in the BINGO cohort were analysed together, we saw a significant difference between infants receiving MF and the other two feeding groups.

**Figure 6:**
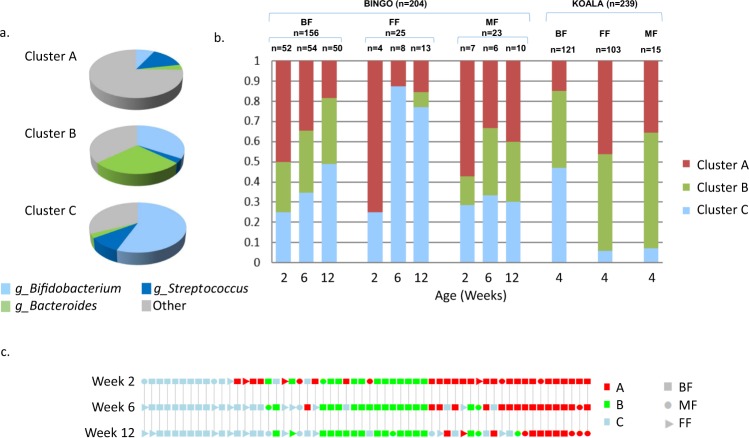
DMM clustering of samples on the basis of faecal microbiota composition at genus level; (**a**) Average relative abundance of microbial groups characteristic to individual cluster A, B and C; (**b**) Fraction of samples from infants receiving different types of feeding (BF, breastfeeding; FF, formula feeding; MF, mixed feeding) within each cluster category; (**c**) Temporal evolution of cluster assignment for infants in the BINGO study cohort, indicating cluster type (red - cluster A, green - cluster B, blue - cluster C), and type of feeding at each time point (square - BF, circle - MF, triangle - FF).

We observed a large variation in microbiota composition between individual infants in both studies, independent of feeding or delivery mode. We used Dirichlet Multinomial Mixtures (DMM) modelling^22^ to assign samples into three clusters based on the relative abundance of the microbial groups at genus level of classification (Fig. 6a). The clustering was performed independently for both studies. There were some minor differences in the average relative contribution of individual taxa between the clusters A, B and C, from each sample sets, but the overall pattern within each cluster type was preserved (Fig. S3). Cluster A contained samples with mixed microbial composition, low relative abundance of *Bifidobacterium* and relatively high proportion of *Streptococcus* and other microbial groups. Cluster B showed high relative abundance of both *Bifidobacterium* and *Bacteroides*, whereas in cluster C *Bifidobacterium* was the dominating genus (Fig. 6a). The same cluster pattern was visible when samples were divided into subgroups based on the infants’ age or delivery mode (data not shown). Infant age and feeding mode were associated with cluster assignment of samples in the BINGO cohort. At two weeks of age, 50% of all samples from BF infants, 75% from FF and 57% of MF infants clustered in group A (Fig. 6b), but as the infants aged their faecal microbiota composition gradually became dominated by *Bifidobacterium* (clusters B and C). This gradual transition pattern was clear in BF infants, however, it was distorted in infants who received formula, either as a sole source of food or as supplementary feeding (Fig. 6b). In the subset of the individual infants (n = 60) from the BINGO cohort, where samples from all three time points were available, infants were more likely to stay within the same cluster between two and 12 weeks of age, and if they switched to a different cluster group the change was towards the *Bacteroides*/*Bifidobacterium* or *Bifidobacterium* rich clusters B and C (Fig. 6c). While all infants that received mixed feeding remained in cluster A, all infants who changed from breastfeeding to formula also switched from clusters A or B into cluster C. In the KOALA cohort, cluster assignment of BF infants showed a similar result to that of the BINGO cohort at six weeks. However, an opposite trend was found for FF and MF infants, where over 90% of infants could be categorized within clusters A or B, showing low to moderate relative abundance of faecal *Bifidobacterium*.

Clustering of the 186 samples from the BINGO cohort corresponding to the 66 vaginally delivered infants led to the same cluster structure. Similar results were obtained when clustering was restricted to the 211 vaginally delivered infants from the KOALA cohort. Restriction of the analysis to the samples from C-section born infants led to no reliable clusters due to the low number of samples (n = 20 in BINGO, n = 28 in KOALA). Cluster assignments of samples grouped for other characteristics (delivery place, infant sex or weight at birth) were inspected and compared with overall study distribution, but no significant associations were found (p > 0.05).

In order to examine the effect of the time gap between the KOALA and BINGO cohorts, and the differences in infant ages and DNA extraction protocols, we compared microbiota profiles of two subsets of samples obtained from vaginally delivered, healthy BF infants at six weeks (BINGO cohort: n = 51, girls/boys ratio = 23/28, *mean* age = 6.1, *median* age = 6.1, *SD* = 0.20) and at four to six weeks old (KOALA cohort: (n = 97, girls/boys ratio = 48/49, *mean* age = 4.5 wk, *median* age = 4 wk, *SD* = 0.59). RDA analysis revealed that when infant age and gender were treated as covariates, the cohort time could explain 1.24% of residual variation in the microbiota (FDR = 0.01). In the BINGO cohort there was a higher relative abundance of *Actinomyces, Streptococcus, Enterococcus, Bifidobacterium*, Peptostreptococcaceae *g_Incertae_Sedis*, and unidentified genera within families Enterobacteriaceae and Actinomycetaceae (FDR < 0.05), whereas in the KOALA cohort there was a higher relative abundance of *Lactococcus, Leuconostoc, Clostridium* and unidentified genus within family Streptococcaceae (Kruskal-Wallis analysis).

## Discussion

The current study examined faecal microbiota composition in healthy infants sampled at two, six and 12 weeks of age from the BINGO study cohort (year 2015–2016) and infants sampled at approximately one month of age from the KOALA study cohort (year 2002–2003). All infants received either commercially available formulas, breastmilk or mixed feeding. At the time samples from the KOALA cohort were collected, infant formulas enriched with prebiotics were available on the Dutch market only to a limited extent. It is important to note that a number of other factors such as lifestyle, maternal diet, infant age, delivery and rearing practices, climate, and many other environmental factors have changed between the time the samples from the KOALA and BINGO cohorts were collected. These factors might also have influenced the composition of infant microbiota, in addition to the effects due to different feeding modes. In fact, we could detect difference in the microbiota of the vaginally delivered, exclusively BF infants of similar ages from the two study cohorts. Thus, to reduce bias associated with using data from two different study cohorts we focused our analyses on the within-cohort comparisons. Using this approach, we showed that faecal microbiota composition of infants fed modern types of formulas containing prebiotics was more similar to faecal microbiota of BF infants. This trend was not observed in FF infants receiving unsupplemented formulas available in years 2002–2003. The bifidogenic effect of prebiotic fortified formulas was not observed in MF infants.

The majority of infant formulas available on the Dutch market nowadays are supplemented with GOS and/or FOS, but new innovations are appearing, such as chicory oligofructose(FOS):lcFOS mixes used in hypoallergic infant formulas. Previous studies showed that the mixture of GOS and/or FOS stimulated growth of *Bifidobacterium* in preterm^23–25^ and term infants^26–28^, and that GOS/FOS supplementation also resulted in a metabolic activity of the colonic microbiota, as measured by SCFA and faecal pH, that was similar to the metabolic activity in BF controls^26,28^. However, many of the microbiological data in these earlier studies were obtained using traditional culture- or molecular probe-based methods, which were often limited in their accuracy or the scope of microbial detection^29^. Here, we have compared samples from two study cohorts obtained more than ten years apart. The use of next generation sequencing in our study allowed us to gain a more comprehensive insight into microbial composition and dynamic patterns at a microbial community level.

In both studies unconstrained multivariate analysis revealed a clear separation of samples relating to the feeding mode (Fig. 3). This is in agreement with a number of earlier studies which showed distinctively different faecal microbial profiles when comparing BF, FF and MF infants^29–31^. We found that in both study cohorts feeding mode was associated with faecal microbiota composition (Fig. 4), and that bacterial diversity and richness in BF infants was significantly lower than in FF infants, except for the six week old infants from the BINGO cohort for which no difference in diversity was observed (Fig. 2). Diversity evaluates both the number of species and the evenness of their distribution. Earlier studies showed that formula feeding was associated with higher faecal microbial diversity, and more adult like microbiota composition^31,32^ and microbiota activity^33^. Recently, higher microbial diversity at four months of age (“premature maturation”) has been associated with an increased risk for developing Celiac Disease later in life^34^. The authors concluded that colonisation dynamics with gradual establishment of complex microbial communities might be important in development of immune tolerance and thus, reduced disease risk. In contrast, higher microbial diversity and high abundance of butyrate producing bacteria in infants around weaning age (6–9 mo.) was associated with alleviating symptoms of atopic eczema, which also supports the idea that the timing in microbiota development might play an important role in immune programming^35^. In the BINGO cohort, FF infants showed an overall higher number of genus level taxa in their faeces in all age groups as estimated by the Chao1 index. This difference was most prominent at six weeks (31.5, *SD* = 8.8 in FF and 22.5 *SD* = 6.5 in BF; Fig. 2b). Yet, the evenness, or relative contribution of each bacterial group, was similar between BF and FF in the BINGO cohort (Fig. 1), possibly due to more comparable relative abundance of the main groups in response to prebiotics^36^. Despite the similarities, the relative abundance of 12 genus level groups differed significantly between BF and FF infants in the BINGO study cohort. It should be noted, however, that the differences were mainly in the low abundance taxa which accounted for a very small fraction of the total bacteria in the community (Fig. 1). In contrast, in the KOALA cohort 19 genus level taxa varied significantly, and the differences in both the species diversity and species richness were significant between FF and BF groups (Fig. 2c,d). Together these differentially abundant taxa in the KOALA cohort accounted for more than 60% of the total bacteria in the faeces of either BF or FF infants.

Despite the differences in the type of formula used, in both the BINGO and the KOALA cohorts FF was associated with a significant increase in relative abundance of *Akkermansia, Enterococcus*, Peptostreptococcaceae *Incertae Sedis* and Erysipelotrichaceae *Incertae Sedis*. Higher levels of these microbial groups in FF infants have been reported previously^7,31,37^. Interestingly, a similar effect was observed in piglets that were fed dairy milk-based formula and showed significantly higher levels of both *Akkermansia* and *Enterococcus*, indicating a possible effect of dairy milk compounds that are present in infant formulas^38^.

Our results showed few important differences in the faecal microbial composition between the BINGO and the KOALA cohorts. *Clostridium* and *Escherichia-Shigella*, both of which include important pathogens, showed higher relative abundance in the FF infants from the KOALA cohort, but not in the BINGO cohort, when compared to the levels found in the corresponding BF groups. Another important difference pertains central bacterial groups, such as *Bifidobacterium* and *Lactobacillus*, whose relative abundance was reduced in the FF group from the KOALA cohort, but not in the BINGO cohort, as compared to BF infants from their corresponding cohorts. These results are in line with earlier findings on the microbiota composition changes due to prebiotic fortification used in formulas^36^. Earlier culture-dependent studies based on colony counts and molecular based methods reported that GOS/FOS stimulated growth of bifidobacteria^23^ and lactobacilli, and led to reduction of *Escherichia coli, Clostridium* species and other clinically relevant pathogens^39^. We showed that prebiotic supplementation resulted in an increase in relative abundance of specific microbial species (or strains) of *Bifidobacterium* OTU L2 which was also the most predominant bifidobacterial OTU detected in the healthy BF infants (Fig. S1). Based on these findings we can conclude that the formulas used by the participants of the BINGO cohort appear to be better in mimicking the beneficial properties of human milk with respect to the stimulation of GI tract microbiota that is similar to that found in healthy BF infants (Fig. 1). In contrast to the earlier described intervention studies^19,40^, these differences have now been shown by comparing data from two population-based studies. In addition, studies on bifidobacteria and lactobacilli reported that prebiotic formulas and breastmilk may stimulate the same bacterial groups, but as it was shown for *Lactobacillus*, they may stimulate different species within the same group^41,42^. Thus, to further support our findings an approach allowing species or strains level identification might be necessary.

One of the main characteristics of the early life GI tract microbial ecosystems are low diversity and low stability^32,43,44^. These characteristics make the microbial ecosystems more vulnerable to environmental disturbance, and may explain the inconsistent patterns of distribution of taxa and high levels of inter-individual variability observed here and in other studies^32,40^. In our study we observed large differences between infants and to disclose any possible more generic patterns in the faecal microbial composition, we applied DMM clustering analysis to all samples from the BINGO and KOALA sets separately. These analyses revealed presence of three distinct clusters with very similar characteristics for the different cohorts (Fig. 6a). Furthermore, these clusters were still present when samples from only vaginally delivered BF infants were used in the modelling. In addition, these clusters were not associated with other characteristics such as sex, birth weight or place of delivery. This implies that other environmental or genetic factors might be driving development of specific microbial assemblages in the infant GI tract^44^, especially that the majority of factors which shape the human gut microbiota still remain unknown^45^. One such factor might be the composition of breastmilk from the individual mother-infant pairs. Earlier studies showed that the composition of breastmilk varies between individual mothers and across lactation stage with regard to human milk oligosaccharide (HMO) content and composition^46^, milk microbiota and other breastmilk factors which could influence GI colonisation^47^.

The large inter-individual variation in faecal microbial composition of infants has been noted before in a number of studies, but none of them indicated existence of enterotypes in infants^2,5,48^. Only recently, it has been suggested that three compositionally distinct human neonatal gut microbiota (NGM) profiles might be present^48,49^ and linked to development of atopy in young infants^48^. However, whether the specific microbial patterns observed in early life are universal, whether they can prompt an individual to develop a given adult enterotype, or if they predispose an infant to any health conditions, remains to be investigated in future longitudinal studies.

The longitudinal design of the BINGO study allowed us to examine the temporal pattern in the development of microbiota in infants from two to 12 weeks of age. Earlier studies indicated that the pattern of microbiota development is non-random^3,50^ and is correlated with the ability of an infant to effectively digest breastmilk HMOs, as the proportion of HMO consuming *Bifidobacterium* and *Bacteroides* increase^51^. We were interested in the relationship between each of the aforementioned clusters, infant age and feeding mode (Fig. 6b). In the BF group there was a clear directional trend, where faecal microbiota gradually transitioned from the “mixed” cluster A to the *Bifidobacterium* dominated clusters B and C as infants got older. This pattern was different in infants receiving prebiotic formula, where exclusive formula feeding, or a change from breastfeeding to formula were associated with an accelerated shift in microbial community composition to cluster C, already at six weeks of age. This suggests that the establishment of a *Bifidobacterium* dominated ecosystem might be linked with the age of an infant or with breastmilk properties, whereas in the FF infants this maturation of the GI tract microbiota was shaped and accelerated by the prebiotic(s) included in the formulas, although this observation should be confirmed using larger sample sizes in the FF group at each time point. In contrast, the mixed feeding was associated with delayed microbiota development, with more infants at 12 weeks of age falling into the mixed microbiota cluster A, as compared to infants from the BF group (Fig. 6b). This mixed state was also characteristic to FF and MF infants receiving formulas with no prebiotics in the KOALA study. The clinical consequences of the timing of colonization with e.g. *Bifidobacterium* are still largely unknown, but a few studies up to date have implied that colonisation dynamics might affect immune programing and the health outcomes later in life^34,40^. The complex and dynamic structure of the human milk could be an important driving force shaping the GI microbiota in BF infants, and possibly play a role in the formation of the cluster groups observed in our data. Given that the amount of HMO’s in maternal milk changes over the first months of life, a way of avoiding the possibly unfavourable acceleration in microbial colonization related to FF would be to design formulas which would mimic the natural changes in HMO’s by varying the amounts of prebiotics for infants of different ages.

Finally, our findings suggest that the use of new formulas in combination with breastmilk feeding was associated with more mixed microbiota composition and showed lower bifidogenic effect, thus implying a possible interference between the components of breastmilk and formula. A similar shift towards FF microbiota pattern in infants receiving MF has been noted in the past^52^. Unfortunately, in both cohorts the MF groups were small (23 BINGO samples, 15 KOALA samples), preventing us from drawing strong conclusions. However, this mode of feeding represents a highly realistic scenario in today’s infant nutrition and thus, should be further investigated using a larger study cohort. Such a study would require a detailed knowledge of breastmilk composition, as well as information on the types of formulas that the infants received to enable detailed assessment of the effect of each component.

## Materials and Methods

### Formula and prebiotics

All formula fed (FF) infants received commercially available formulas purchased in the Netherlands between 2015–2016 (BINGO cohort), and between 2002–2003 (KOALA cohort). The prebiotic fortified infant formulas used in BINGO cohort typically contain scGOS (0.24–0.50 g/100 ml), or mixtures scGOS:lcFOS (9:1); 0.6 g/100 ml^18,53^. In the KOALA cohort 17 of 103 FF infants were confirmed to receive formulas containing scGOS or scGOS:lcFOS, while the remaining infants were fed traditional formulas with no added prebiotics.

### Study description

The analyses described here are part of the BINGO and KOALA birth cohort studies. All infants included in this analysis were born at term (after 37 weeks of gestation), healthy, from singleton pregnancies, had no congenital abnormalities related to growth, and none of the infants received oral or systemic antibiotic treatment during the study period. The BINGO (Dutch acronym for Biological Influences on Baby’s Health and Development) cohort is an ongoing longitudinal study investigating prenatal predictors of infant health and development. This study was approved by and carried in accordance to the ethical committee of the Faculty of Social Sciences of the Radboud University [ECSW2014-1003-189]. Expectant mothers in the BINGO cohort signed up via the project’s website, or folders that were handed out in midwife practices, pregnancy courses, and baby stores in the region Arnhem-Nijmegen (the Netherlands). Exclusion criteria were maternal drug use, regular alcohol consumption, and insufficient knowledge of the Dutch language. Infant faecal samples were collected between years 2015–2016, from a diaper by the mothers within a period of 48 h, when infants were two, six and 12 week old using a sterile stool vial (80 × 16.5 mm; cat#:80.623.022; Sarstedt; Nümbrech, Germany). The mothers were asked to immediately store the samples in their home freezers (i.e., fresh frozen collection) until collected by the researcher. After collection, samples were stored at −80 ^o^C until further processing and analysis.

The design, selection criteria and faeces collection procedure of the KOALA Birth Cohort Study (Dutch acronym for: Child, Parents and Health: Lifestyle and Genetic Constitution) have been described elsewhere, and the study was approved by and carried in accordance with the Ethics Committee of the University Hospital of Maastricht^54,55^. In brief, the KOALA study included two recruitment groups of healthy pregnant women in the South of the Netherlands. The first group (n = 2343) was characterised by a conventional lifestyle, whereas families included in the second group (n = 491) were considered to have an alternative lifestyle that could involve dietary habits (vegetarian, organic), child-rearing practices and/or low use of antibiotics, and were recruited through alternative channels, such as posters in organic food shops, anthroposophic doctors and midwives. Infant faecal samples were collected between years 2002–2003, by the parents at approximately one month postpartum by removing a sample from a diaper into a sterile tube, refrigerated, and sent to the lab by post within one day after collection. Samples were stored at −80 ^o^C in peptone glycerol solution (10 g/L peptone water in 20 v/v% glycerol) at 1 g of faeces in 9 mL of solution until further processing and analysis.

In both studies, parents were asked to sign an informed consent and complete a questionnaire including information regarding infant’s diet (breastmilk only, formula only, or mixed feeding), and the type of infant formula used. The number of samples from each study cohort and relevant information on infant characteristics and feeding mode are summarised in Table 1. In the KOALA cohort the information about feeding mode referred to the time from birth until sample collection, whereas in the BINGO cohort it referred to feeding mode during each time interval, i.e. from birth to the first time point, and between subsequent time points.

**Table 1:** Sample (BINGO), and infant (KOALA) characteristics (N = 443).

BINGO	Total N = 204 samples (77 infants)		
Feeding	Breastfed (n = 156)	Formula fed (n = 25)	Mixed fed (n = 23)
2 weeks	52	4	7
6 weeks	54	8	6
12 weeks	50	13	10
Delivery mode			
Vaginal:	139	23	19
C-section:	14	2	4
No record:	3	0	0
Gender			
Male:	84	8	14
Female:	69	17	9
No record:	3	0	0
Gestation (weeks) Mean ± SEM ± SD	39.78 ± 0.20 ± 1.59	39.82 ± 0.35 ± 1.30	39.58 ± 0.39 ± 1.64
Birth weight (g) Mean ± SEM ± SD	3556 ± 56.21 ± 439	3551 ± 107.52 ± 402	3562 ± 114.27 ± 485
Age at collection (d) Mean ± SEM ± SD			
2 weeks	2.10 ± 0.04 ± 0.25	2.07 ± 0.07 ± 0.14	2.08 ± 0.03 ± 0.08
6 weeks	6.14 ± 0.03 ± 0.20	6.23 ± 0.17 ± 0.06	6.17 ± 0.07 ± 0.17
12 weeks	12.30 ± 0.05 ± 0.36	12.05 ± 0.08 ± 0.28	12.09 ± 0.06 ± 0.19
Health at collection			
Sick*:	12	1	2
Not Sick:	139	24	21
Unknown:	5	0	0
**Maternal age**	**n = 64	**n = 13	**n = 18
Mean ± SEM ± SD	32 ± 0.46 ± 3.7	30 ± 1.00 ± 3.6	32 ± 0.92 ± 3.92
Maternal antibiotics in last trimester	yes (n = 3)	none	none
**KOALA**	**Total N = 239 samples (239 infants)**		
Feeding	Breastfed (n = 121)	Formula fed (n = 103)	Mixed fed (n = 15)
Delivery mode			
Normal vaginal:	100	80	12
Assisted vaginal:	11	4	1
C-section:	10	16	2
No record:	0	3	0
Delivery place			
Home:	72	36	2
Hospital:	49	63	13
No record:	0	4	0
Gender			
Male:	62	61	10
Female:	59	42	5
Gestation (weeks)			
Mean ± SEM ± SD	40.24 ± 1.2 ± 1.2	39.85 ± 0.1 ± 1.3	39.91 ± 0.47 ± 1.8
Birth weight (g)			
Mean ± SEM ± SD	3651 ± 43.48 ± 476	3505 ± 44.05 ± 445	3543 ± 71.27 ± 257
Age at collection (d)			
Mean ± SEM ± SD	32.56 ± 0.5 ± 5.4	31.39 ± 0.49 ± 4.9	35.67 ± 1.3 ± 4.9
Health at collection			
Sick:	3	3	0
Not Sick:	118	100	15
Maternal age	**n = 110	**n = 98	**n = 15
Mean ± SEM ± SD	33 ± 0.32 ± 3.4	33 ± 0.43 ± 4.0	35 ± 0.95 ± 3.7
Maternal antibiotics during last month of pregnancy	yes (n = 3)	yes (n = 3)	none

*Include: common cold, cramps, thrush, reflux or diarrhoea as reported by parents at time of sample collection.

**n Indicates the number of mothers for which the data was available.

A total of 443 faecal samples were analysed: 204 samples from 77 infants from the BINGO cohort, and 239 samples from the KOALA cohort infants (Table 1). In the BINGO cohort eight children were born via C-section and 66 children were born vaginally (185 faecal samples). The proportion of samples from C-section infants in each feeding mode group was 0.08 for breastfed (BF) and for FF infants, and 0.17 in the mixed fed (MF) group. In total 60 infants could be followed at all three time points. In the KOALA cohort 239 samples were analysed for microbiota composition from 239 infants at approximately one month of age (mean age of all infants = 31 days, *SD* = 5). Of all infants, 121 were BF, 103 were FF and 15 were MF. The proportion of samples from C-section infants (n = 28) in each feeding mode group was 0.08 in BF, 0.13 in FF, and 0.12 in MF.

### DNA extraction, library preparation and sequencing

In the KOALA cohort, the total DNA was extracted from the stool samples as previously described^8^, using the double bead-beating procedure followed by QIAamp DNA stool mini kit (Qiagen, Hilden, Germany) according to the manufacturer’s instructions. In the BINGO cohort, total DNA extraction was done using the double bead-beating procedure with 0.1−0.15 g of faecal sample and 350 µL STAR buffer as previously described^56^. The V4 region of 16 S ribosomal RNA (rRNA) gene was amplified in duplicate PCR reactions, each in a total volume of 50 µL and containing 5–20 ng (KOALA) or 20 ng (BINGO) of template DNA^56^ using uniquely barcoded primers 515F-n (5′-GTGCCAGCMGCCGCGGTAA-) and 806R-n (5′-GGACTACHVGGGTWTCTAAT)^56^.

### Data analysis

The 16S rRNA sequencing data was processed and analysed using the NG-Tax analysis pipeline^21^. In brief, libraries were filtered to contain only read pairs with perfectly matching barcodes that were subsequently used to separate reads by sample. Operational taxonomic units (OTUs) were assigned using an open reference approach and SILVA_111_SSU 16S rRNA gene reference database^57^. Diversity analyses were carried out in QIIME on rarefied data with OTU cut-off of 2500^58,59^, and multivariate analysis was done in Canoco5^60^. PCA and RDA were performed using the log transformed genus level relative abundances data obtained from NG-Tax. RDA is a multivariate linear regression model where several response parameters can be related to a set of environmental (explanatory) variables. Statistical significance was assessed in Canoco5 under the full model using the Monte Carlo permutation test with 499 random permutations. Alpha diversity indices (Shannon, Chao1, and PD Whole Tree) for each sample were calculated in QIIME using genus level (L6) data obtained with NG-Tax. Alpha diversity index group comparisons were done in GraphPad (GraphPad Prism version 5.00 for Windows, GraphPad Software, San Diego, CA, USA, www.graphpad.com). When values within a group used in the analysis did not pass the Shapiro-Wilk normality test, the Kruskal–Wallis analysis was used to compare diversity indexes. If the values were normally distributed in all groups, Two-Way ANOVA analysis was used for group comparison. Statistical differences in relative abundance of genus level taxa between three different age groups or feeding modes were assessed with the Kruskal–Wallis test using QIIME. For pairwise comparisons the Wilcoxon test was used when possible, otherwise the Kruskal–Wallis test was applied (QIIME). The proportion of false positives was controlled by accounting for the False Discovery Rate (FDR)^61^. The significance cut-off was set at p < 0.05, and when multiple test correction was applied, an FDR ≤ 0.05 was chosen as threshold. Clusters were identified based on genus level microbial abundance data using Dirichlet Multinomial Mixture (DMM) modelling as described previously^22^. The number of Dirichlet components was selected by inspection of the fit of the model to the count data for varying numbers of components (1 to 7). Goodness of fit was assessed using the Laplace and the Akaike information criteria. Finally, each sample was assigned to the component for which it had the largest fitted value. These analyses were performed in R (version 3.3.1) using the DirichletMultinomial R package^62^.

## Supplementary information

Supplementary Tables and Figures

## Data Availability

KOALA data sets cannot be made publicly available due to data confidentiality and the potential to identify individual study participants from the data. Data are available to the research community through the Dataverse repository (URL hdl:10411/CEGPGR) upon request. BINGO data sets currently cannot be made publicly available due to the data being part of an ongoing longitudinal study. Parts of the data are available to the research community for scientific collaborations upon request to Prof. Dr. C. de Weerth at: Radboud University, Department of Developmental Psychology, Montessorilaan 3, 6525 HR Nijmegen, The Netherlands, e-mail: Carolina.deWeerth@radboudumc.nl.

## References

[1] Collado MC, Rautava S, Aakko J, Isolauri E, Salminen S (2016). Human gut colonisation may be initiated in utero by distinct microbial communities in the placenta and amniotic fluid. Scientific reports.

[2] Valles Y (2014). Microbial succession in the gut: directional trends of taxonomic and functional change in a birth cohort of Spanish infants. PLoS genetics.

[3] Koenig, J. E. *et al*. Succession of microbial consortia in the developing infant gut microbiome. *PNAS* **108** (2010). 10.1073/pnas.1000081107 PMC3063592 20668239

[4] Fouhy F, Ross RP, Fitzgerald GF, Stanton C, Cotter PD (2012). Composition of the early intestinal microbiota: knowledge, knowledge gaps and the use of high-throughput sequencing to address these gaps. Gut microbes.

[5] Madan JC, Farzan SF, Hibberd PL, Karagas MR (2012). Normal neonatal microbiome variation in relation to environmental factors, infection and allergy. Current opinion in pediatrics.

[6] Madan JC (2016). Association of Cesarean Delivery and Formula Supplementation With the Intestinal Microbiome of 6-Week-Old Infants. JAMA pediatrics.

[7] Azad MB (2013). Gut microbiota of healthy Canadian infants: profiles by mode of delivery and infant diet at 4 months. CMAJ: Canadian Medical Association journal = journal de l’Association medicale canadienne.

[8] Penders J (2006). Factors Influencing the Composition of the Intestinal Microbiota in Early Infancy. Pediatrics.

[9] Le Huerou-Luron I, Blat S, Boudry G (2010). Breast- v. formula-feeding: impacts on the digestive tract and immediate and long-term health effects. Nutrition research reviews.

[10] Stevens EE, Patrick TE, Pickler R (2009). A history of infant feeding. The Journal of perinatal education.

[11] Fomon SJ (2001). Infant Feeding in the 20th Century: Formula and Beikost. Journal of Nutrition.

[12] Cabrera-Rubio R (2012). The human milk microbiome changes over lactation and is shaped by maternal weight and mode of delivery. The American journal of clinical nutrition.

[13] Barile D, Rastall RA (2013). Human milk and related oligosaccharides as prebiotics. Current opinion in biotechnology.

[14] Kunz, C. & Egge, H. In *Prebiotics and probiotics in human milk: origins and functions of milk-borne oligosaccharides and bacteria*. (eds M. K. McGuire, M. A. McGuire, & L. Bode) Ch. 1: From Bifidus Factor to Human Milk Oligosaccharides: A Historical Perspective on Complex Sugars in Milk, 3–16 (Academic Press 2016).

[15] Stark PL, Lee A (1982). The microbial ecology of the large bowel of breast-fed and formula-fed infants during the first year of life. J Med Microbiol.

[16] Kunz C, Rudloff S (1993). Biological functions of oligosaccharides in human milk. Acta Paediatr.

[17] Efsa Panel on Dietetic Products Nutrition Allergies. Scientific Opinion on the essential composition of infant and follow‐on formulae. *EFSA Journal* **12**, 10.2903/j.efsa.2014.3760 (2014).

[18] Allergies, E. P. O. D. P. N. Commission Delegated Regulation 2016-127 suppl 609-2013 (infant & young child formula). *EFSA Journa*l (2015).

[19] Vandenplas Y, Zakharova I, Dmitrieva Y (2015). Oligosaccharides in infant formula: more evidence to validate the role of prebiotics. The British journal of nutrition.

[20] Firmansyah A (2016). Fructans in the first 1000 days of life and beyond, and for pregnancy. Asia Pacific journal of clinical nutrition.

[21] Ramiro-Garcia J (2016). NG-Tax, a highly accurate and validated pipeline for analysis of 16S rRNA amplicons from complex biomes. F1000Research.

[22] Ian Holmes KH (2012). Christopher Quince. Dirichlet Multinomial Mixtures: Generative Models for Microbial Metagenomics. PLoS One.

[23] Boehm G (2002). Supplementation of a bovine milk formula with an oligosaccharide mixture increases counts of faecal bifidobacteria in preterm infants. Arch Dis Child Fetal Neonatal Ed.

[24] Kapiki A (2007). The effect of a fructo-oligosaccharide supplemented formula on gut flora of preterm infants. Early human development.

[25] Srinivasjois R, Rao S, Patole S (2013). Prebiotic supplementation in preterm neonates: updated systematic review and meta-analysis of randomised controlled trials. Clinical nutrition.

[26] Knol J (2005). Colon Microflora in Infants Fed Formula with Galacto- and Fructo-Oligosaccharides: More Like Breast-Fed Infants. Journal of Pediatric Gastroenterology and Nutrition.

[27] Costalos C, Kapiki A, Apostolou M, Papathoma E (2008). The effect of a prebiotic supplemented formula on growth and stool microbiology of term infants. Early human development.

[28] Holscher HD (2012). Effects of Prebiotic-Containing Infant Formula on Gastrointestinal Tolerance and Fecal Microbiota in a Randomized Controlled Trial. Journal of Parenteral and Enteral Nutrition.

[29] Harmsen HJM (2000). Analysis of Intestinal Flora Development in Breast-Fed and Formula-Fed Infants by Using Molecular Identification and Detection Methods. Journal of Pediatric Gastroenterology and Nutrition.

[30] Guaraldi F, Salvatori G (2012). Effect of Breast and Formula Feeding on Gut Microbiota Shaping in Newborns. Frontiers in Cellular and Infection Microbiology.

[31] Wang M (2015). Fecal Microbiota Composition of Breast-fed Infants is Correlated with Human Milk Oligosaccharides Consumed. Journal of pediatric gastroenterology and nutrition.

[32] Yatsunenko T (2012). Human gut microbiome viewed across age and geography. Nature.

[33] Bäckhed F (2015). Dynamics and Stabilization of the Human Gut Microbiome during the First Year of Life. Cell Host & Microbe.

[34] Olivares M (2018). Gut microbiota trajectory in early life may predict development of celiac disease. Microbiome.

[35] Nylund L (2015). Severity of atopic disease inversely correlates with intestinal microbiota diversity and butyrate-producing bacteria. Allergy.

[36] Hojsak, I. & MocicPavic, A. Supplementation of prebiotics in infant formula. *Nutrition and Dietary Supplements*, **69**, 10.2147/nds.s39308 (2014).

[37] Bergström A (2014). Establishment of Intestinal Microbiota during Early Life: a Longitudinal, Explorative Study of a Large Cohort of Danish Infants. Appl. Environ. Microbiol.

[38] Saraf MK (2017). Formula diet driven microbiota shifts tryptophan metabolism from serotonin to tryptamine in neonatal porcine colon. Microbiome.

[39] Knol J (2005). Increase of faecal bifidobacteria due to dietary oligosaccharides induces a reduction of clinically relevant pathogen germs in the faeces of formula-fed preterm infants. Acta paediatrica.

[40] Timmerman HM (2017). Intestinal colonisation patterns in breastfed and formula-fed infants during the first 12 weeks of life reveal sequential microbiota signatures. Scientific reports.

[41] Haarman M, Knol J (2005). Quantitative real-time PCR assays to identify and quantify fecal Bifidobacterium species in infants receiving a prebiotic infant formula. Applied and environmental microbiology.

[42] Haarman M, Knol J (2006). Quantitative real-time PCR analysis of fecal Lactobacillus species in infants receiving a prebiotic infant formula. Applied and environmental microbiology.

[43] Scholtens PAMJ, Oozeer R, Martin R, Amor KB, Knol J (2012). The Early Settlers: Intestinal Microbiology in EarlyLife. Annual Review of Food Science and Technology.

[44] Wopereis H, Oozeer R, Knipping K, Belzer C, Knol J (2014). The first thousand days – intestinal microbiology of early life: establishing a symbiosis. Pediatric Allergy and Immunology.

[45] Falony G (2016). Population-level analysis of gut microbiome variation. Science.

[46] Austin S (2016). Temporal Change of the Content of 10 Oligosaccharides in the Milk of Chinese Urban Mothers. Nutrients.

[47] Walker WA, Iyengar RS (2014). Breast milk, microbiota, and intestinal immune homeostasis. Pediatric Research.

[48] Fujimura KE (2016). Neonatal gut microbiota associates with childhood multisensitized atopy and T cell differentiation. Nature medicine.

[49] Kuang Y-S (2016). Composition of gut microbiota in infants in China and global comparison. Scientific reports.

[50] Favier CF, Vaughan EE, De Vos WM, Akkermans ADL (2002). Molecular Monitoring of Succession of Bacterial Communities in Human Neonates. Applied and environmental microbiology.

[51] De Leoz MLA (2015). Human Milk Glycomics and Gut Microbial Genomics in Infant Feces Show a Correlation between Human Milk Oligosaccharides and Gut Microbiota: A Proof-of-Concept Study. Journal of Proteome Research.

[52] O’Sullivan A, Farver M, Smilowitz JT (2015). The Influence of Early Infant-Feeding Practices on the Intestinal Microbiome and Body Composition in Infants. Nutrition and Metabolic Insights.

[53] Vandenplas Y, Greef ED, Veereman G (2014). Prebiotics in infant formula. Gut microbes.

[54] Kummeling I (2005). Etiology of atopy in infancy: the KOALA Birth Cohort Study. Pediatric allergy and immunology: official publication of the European Society of Pediatric Allergy and Immunology.

[55] Scheepers LE (2015). The intestinal microbiota composition and weight development in children: the KOALA Birth Cohort Study. International journal of obesity.

[56] Gu F (2018). *In Vitro* Fermentation Behavior of Isomalto/Malto-Polysaccharides Using Human Fecal Inoculum Indicates Prebiotic Potential. Molecular nutrition & food research.

[57] Quast C (2013). The SILVA ribosomal RNA gene database project: improved data processing and web-based tools. Nucleic acids research.

[58] Kuczynski, J. *et al*. Using QIIME to analyze 16S rRNA gene sequences from microbial communities. *Current protocols in bioinformatics* **10**, Unit 10 17, 10.1002/0471250953.bi1007s36 (2011). 10.1002/0471250953.bi1007s36 PMC3249058 22161565

[59] Caporaso JG (2010). QIIME allows analysis of high-throughput community sequencing data. Nature methods.

[60] Lepš, P. Š. A. J. Multivariate analysis of ecological data using Canoco 5. In: Canoco support. *Cambridge University Press, UK*., 207–229 (2014).

[61] Colquhoun, D. An investigation of the false discovery rate and the misinterpretation of *p*-values. *Royal Society Open Science* **1**, 10.1098/rsos.140216 (2014). 10.1098/rsos.140216 PMC4448847 26064558

[62] Morgan, M. DirichletMultinomial: Dirichlet-Multinomial Mixture Model Machine Learning for MicrobiomeData. *R package version 1.18.0*. (2017).

