# Supplementary material for: The effect of prebiotic fortified infant formulas on microbiota composition and dynamics in early life
Source: Sci Rep. 2019 Feb 21;9:2434. doi: 10.1038/s41598-018-38268-x (PMC6385197; doi:10.1038/s41598-018-38268-x)
Supplement: Supplementary file 1 — Supplementary Tables and Figures [file 41598_2018_38268_MOESM1_ESM.pdf]

Supplementary information for the manuscript entitled:

The effect of prebiotic fortified infant formulas on microbiota composition and dynamics in early life

Klaudyna Borewicz, Maria Suarez-Diez, Christine Hechler, Roseriet Beijers, Carolina de Weerth, Ilja Arts, John Penders, Carel Thijs, Arjen Nauta, Cordula Lindner, Ellen van Leusen, Elaine E. Vaughan, Hauke Smidt

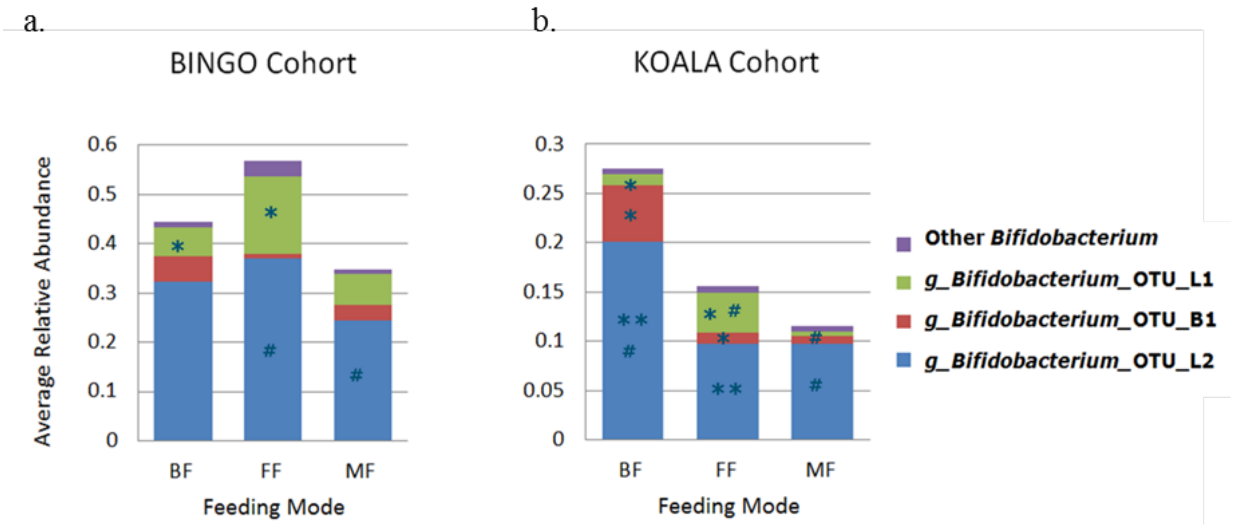

Fig. S1. Relative abundance of bifidobacterial Operational Taxonomic Units (OTUs) in different feeding modes. **a.** Differences of the main *Bifidobacterium* OTUs between formula fed (FF) and breastfed (BF) infants in the BINGO cohort are not significant for OTUs L2 and B1 but significant for OTU L1 ($p<0.05$); **b.** There are significant differences in the main *Bifidobacterium* OTUs between BF and FF infants in the KOALA cohort (OTU L2 FDR<0.05; OTU L1 and B1 $p<0.05$). In both cohorts mixed feeding (MF) resulted in decrease in relative abundance of bifidobacteria, and there was a significant decrease for OTU L2 ($p<0.05$) between FF and MF in the BINGO cohort and between BF and MF in the KOALA cohort, and a significant decrease in OTU L1 in MF compared to FF in the KOALA cohort. Symbols *, #, ** indicate pairs of taxa that were compared and were significantly different in Kruskal - Wallis test ($p<0.05$)

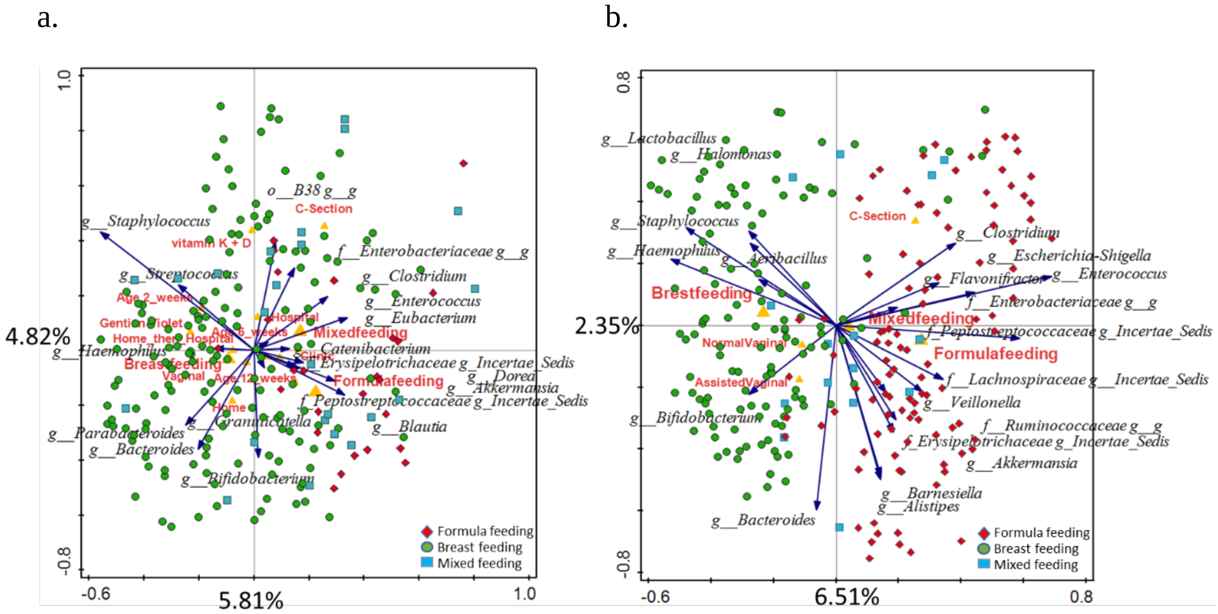

Fig. S2. RDA showing factors with a significant effect on fecal microbiota of infants. Sample are colour coded by feeding mode. Displayed taxa include ten best fitting species, in addition to the microbial groups that differed significantly (Wilcoxon test, FDR<0.05) between BF and FF groups. **a.** BINGO cohort; **b.** KOALA cohort

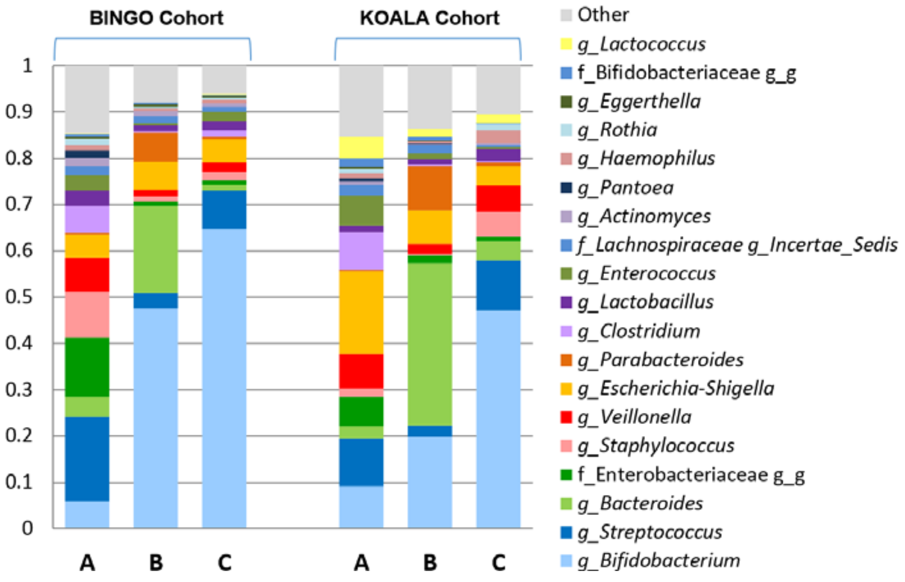

Fig. S3. The average relative contribution of the main bacterial genus-level taxa defining each of three clusters obtained by DMM modelling of fecal microbial composition of infants included in the BINGO and KOALA cohorts. When the taxonomic assignment could not be made at genus level, the lowest classifiable taxonomy assignment is used instead. A - Cluster A characterised by mixed microbial composition; B - Cluster B characterised by high relative abundance of both *Bifidobacterium* and *Bacteroides*; C - Cluster C characterised by high relative abundance of *Bifidobacterium*

Table S1. Average relative abundance of major bacterial taxa detected in BINGO and KOALA cohorts. Microbial groups that significantly differ in relative abundance (Wilcoxon test, FDR<0.05) between BF and FF groups are indicated with * (BINGO) and # (KOALA).

**Study**	**BINGO Cohort**									**KOALA Cohort**		
**Feeding Mode**	**BF**			**FF**			**MF**			**BF**	**FF**	**MF**
**Age (weeks)**	**2**	**6**	**12**	**2**	**6**	**12**	**2**	**6**	**12**	**4**	**4**	**4**
g_Bifidobacterium #	0.3467	0.4614	0.5111	0.4519	0.6218	0.5528	0.2806	0.4321	0.3217	0.3192	0.1718	0.1216
g_Bacteroides	0.0831	0.1212	0.1230	0.0652	0.0225	0.0357	0.0759	0.0877	0.1314	0.2134	0.2316	0.3195
g_Streptococcus	0.0844	0.0711	0.0437	0.0238	0.0314	0.0680	0.1788	0.1223	0.0305	0.0375	0.0454	0.0348
g_Escherichia-Shigella #	0.1108	0.0647	0.0769	0.0380	0.1038	0.0790	0.0431	0.0617	0.0912	0.1185	0.1947	0.2095
f_Enterobacteriaceae *g_g #*	0.1199	0.0524	0.0390	0.0910	0.0061	0.0401	0.0634	0.0919	0.0816	0.0396	0.0555	0.0126
f_Lachnospiraceae *Incertae_Sedis #*	0.0390	0.0268	0.0360	0.0229	0.0084	0.0191	0.0416	0.0593	0.0856	0.0188	0.0276	0.0146
g_Lactobacillus #	0.0105	0.0383	0.0271	0.0480	0.0210	0.0278	0.0201	0.0337	0.0564	0.0228	0.0050	0.0204
g_Clostridium #	0.0277	0.0162	0.0290	0.0167	0.0275	0.0249	0.0677	0.0422	0.0392	0.0256	0.0318	0.0076
*g_Enterococcus #**	0.0223	0.0133	0.0090	0.0042	0.0295	0.0583	0.0507	0.0237	0.0146	0.0020	0.0194	0.0181
*g_Blautia **	0.0002	0.0035	0.0032	0.0245	0.0523	0.0316	0.0468	0.0032	0.0486	0.0053	0.0037	0.0232
g_Veillonella #	0.0237	0.0205	0.0321	0.0167	0.0128	0.0149	0.0260	0.0118	0.0408	0.0274	0.0504	0.0508
*g_Staphylococcus #**	0.0683	0.0136	0.0028	0.0257	0.0035	0.0019	0.0535	0.0018	0.0003	0.0218	0.0006	0.0011
g_Pantoea	0.0029	0.0128	0.0009	0.1201	0.0000	0.0020	0.0006	0.0012	0.0031	0.0015	0.0040	0.0001
*g_Parabacteroides **	0.0131	0.0182	0.0136	0.0006	0.0002	0.0003	0.0269	0.0011	0.0111	0.0668	0.0316	0.0904
f_Erysipelotrichaceae *Incertae_Sedis #**	0.0032	0.0036	0.0050	0.0230	0.0027	0.0089	0.0072	0.0000	0.0101	0.0074	0.0109	0.0098
g_Actinomyces	0.0085	0.0225	0.0043	0.0025	0.0009	0.0015	0.0020	0.0070	0.0026	0.0023	0.0005	0.0212
g_Collinsella	0.0014	0.0004	0.0006	0.0000	0.0314	0.0013	0.0000	0.0000	0.0032	0.0011	0.0008	0.0009
f_Peptostreptococcaceae *Incertae_Sedis #**	0.0001	0.0003	0.0006	0.0019	0.0057	0.0115	0.0013	0.0000	0.0037	0.0001	0.0040	0.0018
f_Lachnospiraceae *g_g*	0.0069	0.0052	0.0004	0.0000	0.0006	0.0005	0.0002	0.0008	0.0071	0.0015	0.0010	0.0000
*g_Haemophilus #**	0.0041	0.0057	0.0055	0.0000	0.0000	0.0000	0.0008	0.0000	0.0005	0.0098	0.0004	0.0062
*g_Catenibacterium **	0.0000	0.0000	0.0000	0.0090	0.0050	0.0001	0.0000	0.0000	0.0000	0.0010	0.0000	0.0000
*g_Akkermansia #**	0.0000	0.0000	0.0000	0.0000	0.0011	0.0050	0.0000	0.0008	0.0003	0.0000	0.0225	0.0008
*g_Dorea **	0.0000	0.0001	0.0000	0.0016	0.0026	0.0023	0.0000	0.0000	0.0000	0.0000	0.0002	0.0000
g_Flavonifractor #	0.0000	0.0020	0.0020	0.0014	0.0000	0.0002	0.0002	0.0000	0.0000	0.0034	0.0047	0.0000
g_Halomonas #	0.0003	0.0005	0.0002	0.0000	0.0000	0.0011	0.0002	0.0012	0.0002	0.0007	0.0000	0.0001
f_Ruminococcaceae *g_g #*	0.0000	0.0003	0.0001	0.0017	0.0002	0.0005	0.0000	0.0000	0.0000	0.0033	0.0044	0.0003
*g_Eubacterium **	0.0000	0.0000	0.0000	0.0014	0.0004	0.0000	0.0000	0.0000	0.0000	0.0004	0.0002	0.0000
g_Aeribacillus #	0.0001	0.0002	0.0001	0.0000	0.0000	0.0005	0.0000	0.0005	0.0000	0.0004	0.0000	0.0001
g_Alistipes #	0.0002	0.0001	0.0001	0.0000	0.0000	0.0001	0.0000	0.0003	0.0003	0.0013	0.0058	0.0029
g_Barnesiella #	0.0000	0.0000	0.0002	0.0000	0.0003	0.0000	0.0002	0.0000	0.0000	0.0018	0.0145	0.0031
*g_Granulicatella**	0.0000	0.0000	0.0001	0.0000	0.0000	0.0004	0.0000	0.0000	0.0001	0.0000	0.0000	0.0000
**TOTAL**	**0.9775**	**0.9750**	**0.9666**	**0.9918**	**0.9918**	**0.9900**	**0.9877**	**0.9840**	**0.9842**	**0.9545**	**0.9432**	**0.9715**
Other taxa	0.0225	0.0250	0.0334	0.0082	0.0082	0.0104	0.0123	0.0160	0.0158	0.0488	0.0615	0.0285

Table S2. Selected V4 16S rRNA gene based OTUs showing a list of matching taxa with the highest total NCBI scores (NCBI Blast on 12/11/2018)

***g*_Bifidobacterium_B1: Total BLAST Score: 262**<br>TACGTAGGGCGCAAGCGTTATCCGGATTTATTGGGCGTAAAGGGCTCGTAGGCGGCTCGTCGCGTCCGGT<br>GCCTGTTCGCTCCCCACGCTTTCGCTCCTCAGCGTCAGTGACGGCCCAGAGACCTGCCTTCGCCATCGGTG
NR_044771.1 *Bifidobacterium bifidum* strain KCTC 3202 16S ribosomal RNA, partial sequence<br>NR_117505.1 *Bifidobacterium bifidum* strain KCTC 3202 16S ribosomal RNA gene, partial sequence<br>NR_117764.1 *Bifidobacterium bifidum* strain DSM 20456 16S ribosomal RNA gene, partial sequence<br>NR_113873.1 *Bifidobacterium bifidum* strain NBRC 100015 16S ribosomal RNA gene, partial sequence
***g*_Bifidobacterium_L1: Total BLAST Score: 262**<br>TACGTAGGGTGCAAGCGTTATCCGGAATTATTGGGCGTAAAGGGCTCGTAGGCGGTTCGTCGCGTCCGGT<br>GCCTGTTCGCTCCCCACGCTTTCGCTCCTCAGCGTCAGTGACGGCCCAGAGACCTGCCTTCGCCATTGGTG
NR_036857.1 *Bifidobacterium ruminantium* strain Ru 687 16S ribosomal RNA gene, partial sequence<br>NR_041348.1 *Bifidobacterium tsurumiense* strain OMB115 16S ribosomal RNA gene, partial sequence<br>NR_116746.1 *Bifidobacterium stercoris* strain Eg1 16S ribosomal RNA gene, partial sequence<br>NR_118589.1 *Bifidobacterium stercoris* strain Eg1 16S ribosomal RNA gene, partial sequence<br>NR_114397.1 *Bifidobacterium moukalabense* strain GG01 16S ribosomal RNA gene, partial sequence<br>NR_133982.1 *Bifidobacterium faecale* strain CU3-7 16S ribosomal RNA, partial sequence<br>NR_037115.2 *Bifidobacterium dentium* strain B764 16S ribosomal RNA gene, partial sequence<br>NR_074802.2 *Bifidobacterium adolescentis* strain ATCC 15703 16S ribosomal RNA, complete sequence
***g*_Bifidobacterium_L2: Total BLAST Score: 262**<br>TACGTAGGGTGCAAGCGTTATCCGGAATTATTGGGCGTAAAGGGCTCGTAGGCGGTTCGTCGCGTCCGGT<br>GCCTGTTCGCTCCCCACGCTTTCGCTCCTCAGCGTCAGTAACGGCCCAGAGACCTGCCTTCGCCATTGGTG
NR_043437.1 *Bifidobacterium longum* subsp. *infantis* strain ATCC 15697 16S ribosomal RNA gene, partial sequence<br>NR_037118.1 *Bifidobacterium gallicum* strain P6 16S ribosomal RNA gene, partial sequence<br>NR_040783.1 *Bifidobacterium breve* strain DSM 20213 16S ribosomal RNA gene, partial sequence<br>NR_041875.1 *Bifidobacterium catenulatum* strain DSM 16992 16S ribosomal RNA gene, partial sequence<br>NR_117506.1 *Bifidobacterium longum* strain KCTC 3128 16S ribosomal RNA gene, partial sequence<br>NR_112779.1 *Bifidobacterium kashiwanohense* strain HM2-2 16S ribosomal RNA gene, partial sequence<br>NR_037117.1 *Bifidobacterium pseudocatenulatum* strain B1279 16S ribosomal RNA gene, partial sequence<br>NR_113174.1 *Bifidobacterium stellenboschense* strain AFB23-3 16S ribosomal RNA gene, partial sequence<br>NR_145535.1 *Bifidobacterium longum* subsp. *suillum* strain Su 851 16S ribosomal RNA, partial sequence<br>NR_159261.1 *Bifidobacterium callitrichidarum* strain TRI 5 16S ribosomal RNA, partial sequence
